# Self-cleaning superhydrophobic fly ash geopolymer

**DOI:** 10.1038/s41598-022-27061-6

**Published:** 2023-01-02

**Authors:** Prinya Chindaprasirt, Peerapong Jitsangiam, Pumipat K. Pachana, Ubolluk Rattanasak

**Affiliations:** 1 Department of Civil Engineering, Faculty of Engineering, Sustainable Infrastructure Research and Development Center, Khon Kaen University, Khon Kaen, 40002 Thailand; 2 Academy of Science, Royal Society of Thailand, Dusit, Bangkok, 10300 Thailand; 3 Department of Civil Engineering, Faculty of Engineering, Center of Excellence in Natural Disaster Management, Chiang Mai University, Chiang Mai, 50200 Thailand; 4 Department of Chemistry, Faculty of Science, Burapha University, Chonburi, 20131 Thailand; 5 Center of Excellence on Environmental Health and Toxicology (EHT), OPS, MHESI, Bangkok, 10400 Thailand

**Keywords:** Civil engineering, Surface chemistry

## Abstract

Building materials with hydrophobic surfaces can exhibit increased service life by preventing moisture absorption or diffusion through their surfaces. For concrete used in construction, this hydrophobicity can prevent the corrosion of reinforcing steel bars. Geopolymers are a new cement-free binding material that have been extensively studied to replace Portland cement. However, similar to normal concrete, geopolymers are susceptible to the intake of moisture. This paper presents the fabrication of a superhydrophobic and self-cleaning surface on a fly ash geopolymer as a method to prevent moisture intake. A composite coating of polydimethylsiloxane (PDMS) solution containing dispersed polytetrafluoroethylene (PTFE) or calcium stearate (CS) microparticles was applied by dip-coating to form the hydrophobic surface. Additionally, fly ash was incorporated with the PTFE and CS microparticles to increase surface roughness and reduce material cost. The experimental results showed that the coating containing CS microparticles yielded a hydrophobic surface with a contact angle of 140°, while those containing PTFE microparticles provided a superhydrophobic surface with a contact angle of 159°. The incorporation of fly ash resulted in increased surface roughness, leading to a larger contact angle and a smaller sliding angle. A contact angle of 153° with a sliding angle of 8.7° was observed on the PTFE/fly ash-coated surface. The cleaning process was demonstrated with a test whereby dust was removed by water droplets rolling off the surface. The tested coating exhibited self-cleaning and waterproofing properties and could thus improve the sustainability of materials in building construction.

## Introduction

The surface hydrophilicity or wettability of building materials is one of the most influential parameters affecting their service life. Moisture can absorb into a concrete matrix, resulting in cracks, algal and fungal growth^1^, and corrosion of the reinforcing steel bars^2^. In addition, dust can deposit on building materials, requiring cleaning and resulting in additional maintenance costs. Therefore, the development of superhydrophobic and self-cleaning surfaces for building materials is significant in protecting the matrix from water intake and allowing for easy dust removal by sliding water^3^. Superhydrophobic surfaces have low adhesion force and surface energy, reducing the accumulation of dust particles and facilitating surface cleaning during rainfall^4^. Superhydrophobicity also provides antimicrobial and anticorrosion protection to concrete, thus extending its service life.

A superhydrophobic surface is defined as a surface with static water contact angles greater than 150°^5^. A sliding angle of less than 10° is also required for self-cleaning properties, together with a high contact angle^6^. A low sliding angle is referred to as the “lotus effect” after the superhydrophobic and self-cleaning behavior of lotus leaves^7,8^.

In the construction and textile industries, a silicon-based compound is often used as the water-repelling agent to produce a hydrophobic coating^9–11^. Spraying, dip-coating, and painting are simple and cost-effective techniques for generating superhydrophobic surfaces on building materials^3,12–14^. Due to its low surface energy, polydimethylsiloxane (PDMS) is an important silicon-based compound widely used as a base coating to achieve hydrophobicity^10,11^. However, the use of only PDMS results in poor adhesion to the textile surface and does not provide surface roughness and self-cleaning properties; thus, the resulting material has limited durability upon exposure to adverse environments. Generally, the wettability of solid surfaces is controlled by the chemical composition and geometric features on the surface.

Superhydrophobicity can be achieved by creating a rough surface on substrates with low surface energy^11,15^. Different types of microparticles have been used to create surface roughness, such as titanium oxide^11^, SiO_2_ nanoparticles^10^, and polytetrafluoroethylene (PTFE)^3,13,16^. PTFE has a low surface energy and excellent properties for reducing adhesion and friction. In the construction industry, calcium stearate (CS) has been used as a water-repellent additive to damp-proof concrete. However, a high proportion of CS increases the air content, reducing the density of the concrete^17^. The surface energy of PTFE is 19 mJ/m^2^^18^, whereas that of CS is 23 mJ/m^2^^19^. However, CS is generally selected over PTFE to improve the hydrophobicity of building materials due to its lower cost.

Currently, employing green building materials is widely encouraged. To this end, the utilization of industrial waste ash to reduce the amount of Portland cement in concrete mixtures is one green approach. Industrial waste ashes, which are fly ash (FA), bottom ash, rice husk ash, bagasse ash and palm oil fuel ash, are increasingly employed to partially replace Portland cement in pavement and roadways^20–23^. Geopolymers are cement-free binders prepared by the reaction of silica- and alumina-rich materials with alkali solutions. The preferred source materials are fly ash, slag and metakaolin, and the most commonly used alkali activators are sodium hydroxide and sodium silicate. These solutions react with active silica (SiO_2_) and alumina (Al_2_O_3_) and form a Si–O–Al network^24,25^. It sets and hardens similarly to cement paste. This material has been extensively studied due to its good performance^26,27^.

Similar to cement-based materials, geopolymers can absorb moisture and water because they have porous structures and contain many voids. Therefore, this study proposes the fabrication of superhydrophobic and self-cleaning surfaces on geopolymers. This involves a rough surface coating produced by incorporating PTFE and CS microparticles dispersed in a PDMS binder, in which PDMS binds and fixes the microparticles to the material surface^11^. Researchers have reported the fabrication of hydrophobic surfaces on building materials^3^ and cotton fabrics^28^ with the use of fly ash. Two-step spray coating has been used on building materials^3^ by which epoxy resin solution is first sprayed on the surface followed by the spraying of a fly ash suspension. For cotton fabrics^28^, the hydrophobic surface is prepared by immersing fabrics in a fly ash-suspended solution, drying, and then curing at 120 °C. Then, an OTES is applied to the fly ash-coated cotton fabrics to lower the surface energy. The coating materials developed here could be applied in one step via dip-coating or painted on the surface of building materials. In this study, the dip-coating method was chosen to apply a PDMS composite coating containing dispersed PTFE or CS microparticles, in which FA was also incorporated to provide additional surface roughness and reduce material costs^3^. The contact angles and sliding angles of water droplets on the coating surface were measured to characterize the superhydrophobic and self-cleaning properties. The developed composite coating should also be useful for producing superhydrophobic coatings on other building materials.

## Materials and methods

### Materials

**Figure 1:**
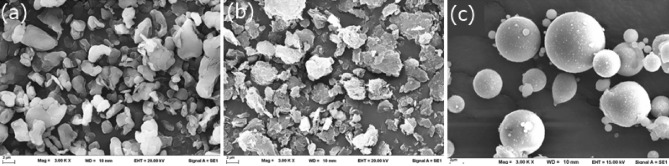
SEM images of the (**a**) PTFE, (**b**) CS, and (**c**) FA particles.

PTFE and CS microparticles were used as water-repellent materials to fabricate superhydrophobic surfaces. Commercial-grade PTFE and CS were purchased from a chemical supplier. The FA was from a BLCP power plant in Rayong Province, Thailand. FA was a byproduct of the pulverized coal combustion process using bituminous coal as a feedstock. It was used to prepare geopolymers and replace water-repellent materials to reduce material costs and provide greater surface roughness^3^. The PTFE and CS particles were white, and the FA was dark brown. The median particle sizes (D50) of PTFE, CS, and FA were 3.3, 8.6, and 13.1 µm, respectively, as measured by a particle size analyzer (Malvern Mastersizer 3000); additionally, their corresponding specific gravity values were 2.1, 1.1 and 2.2, as tested in accordance with ASTM C188-14. The physical properties of these microparticles are shown in Table 1. The morphology of the particles, from scanning electron microscopy (SEM, Leo 1450VP, gold coating), is shown in Fig. 1. The PTFE and CS particles have irregular shapes, whereas the FA particles are spherical due to their rapid solidification while suspended in the hot flue gas.

**Table 1:** Physical properties of the microparticles.

Microparticles	Median particle size (μm)	Specific gravity	Surface energy (mJ/m^2^)
Polytetrafluoroethylene	3.3	2.1	19^18^
Calcium stearate	8.6	1.1	23^19^
Fly ash	13.1	2.2	45^29^

In addition, liquid PDMS with a curing agent, hexane, and sodium silicate solution (Na_2_SiO_3_, 30 wt% SiO_2_, 9 wt% Na_2_O) were used. An 8 M sodium hydroxide solution (8 M NaOH) was prepared from solid NaOH. Fine river sand with a specific gravity of 2.7 and a fineness modulus of 1.9 was used to prepare the geopolymer mortar.

### Preparation of the FA geopolymer mortar

The FA geopolymer mortar was used as the substrate for the coating. The blended alkali solution was prepared with a Na_2_SiO_3_-to-NaOH mass ratio of 2. One hundred grams of FA and 100 g of alkali solution were mixed in a mixing bowl. The mixture was mixed until a uniform paste was obtained. Then, 200 g of fine river sand was added to the paste. The mortar was poured into 2.5 × 5 × 1 cm^3^ silicone molds and cured at 65 °C for 24 h^26^. The hardened geopolymer mortars were demolded and kept in zip-close bags.

### Coating preparation

**Figure 2:**
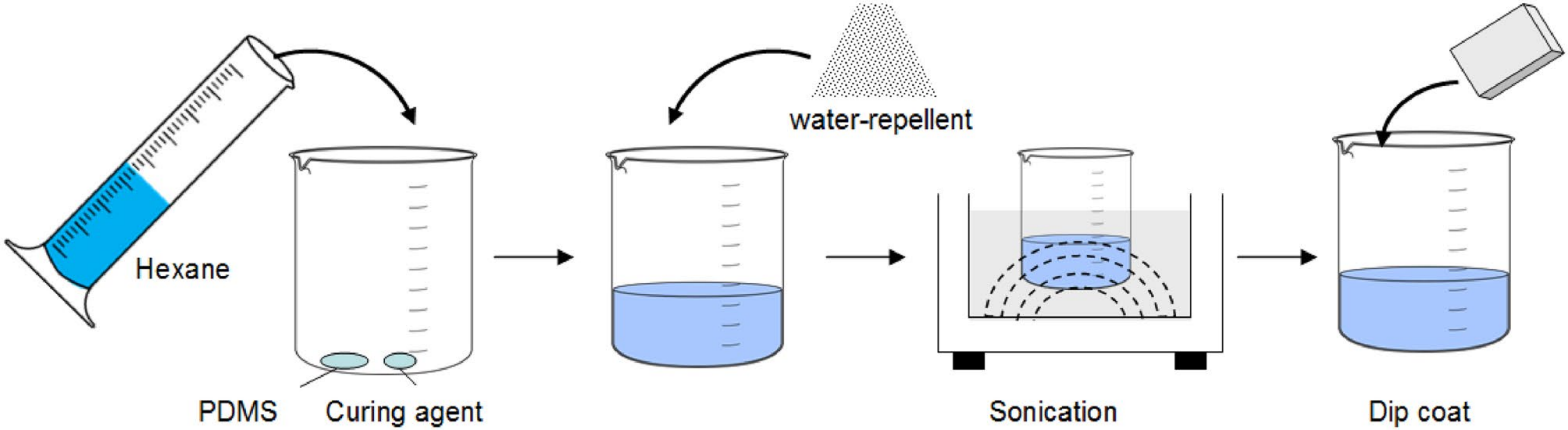
Fabrication of the water repellent-coated superhydrophobic geopolymer.

PDMS is an inexpensive hydrophobic polymer and was used to coat solid surfaces. The PDMS solution was prepared by mixing 0.9 g of the PDMS base with 0.1 g of the curing agent in a beaker. The solution was then diluted with 30 mL of hexane as a thinner^10,15^. The microparticles (PTFE or CS) were thoroughly dry-mixed with FA and then added to the PDMS solution. Sonication was performed on the mixture using an ultrasonic bath for 10 min to disperse the microparticles in the PDMS solution and to allow the formation of the coating precursor^3^. Subsequently, the 28-day geopolymer mortars were dipped in the solution for 30 s and allowed to dry at room temperature (26–30 °C) for 24 h. The proportions of the water-repellent mixtures are shown in Table 2. The thicknesses of the different coatings tested were measured in terms of the surface roughness using SEM imaging and the ImageJ program. This roughness measurement showed the average roughness (Ra). A schematic diagram for the fabrication of the water repellent-coated superhydrophobic geopolymer is shown in Fig. 2.

**Table 2:** Mix proportions of the water-repellent mixtures and Ra values of the coated surfaces.

Samples	Water-repellent material			Coated layer			Ra value (μm)
	CS (g)	PTFE (g)	FA (g)	PDMS (g)	Curing agent (g)	Hexane (mL)	
Control	–	–	–	1	0.1	30	–
CS	1	–	–	1	0.1	30	43.4
CS-F	0.9	–	0.1	1	0.1	30	50.2
PTFE	–	1	–	1	0.1	30	49.9
PTFE-F	–	0.9	0.1	1	0.1	30	55.6

### Characterization of the dip-coated geopolymers

The contact and sliding angles of a water droplet on the coated specimens were measured using a contact angle goniometer (Ossila L2004A1). The results are the average of three tests with a p value less than 0.05 (5%). The microstructure formed on the coated surfaces was also examined by SEM. The SEM images of the coated surface were used to create a 3D surface plot and for surface profile reconstruction using the ImageJ program.

The durability of the coated surfaces was determined by an adhesion test following ASTM C3359-17^30^. Briefly, a grid of 1 mm × 1 mm with six cuts was drawn on top of a coated sample. Subsequently, a piece of pressure-sensitive tape (Scotch tape) was applied over the lattice pattern and then removed. Coating adhesion was evaluated qualitatively on a 0–5 scale.

### Self-cleaning test

The self-cleaning test was performed to determine the ability to remove dust that generally results from the accumulation of smoke particulates or industrial emissions. In this study, bagasse ash was selected to represent dust from industrial emissions and used to determine the self-cleaning ability of the coatings. The ash was obtained from the combustion of bagasse in a sugar factory^31^. The bagasse ash was passed through a No. 40 sieve (0.42 mm opening) as recommended by  ASTM D2132-19^32^ to determine the self-cleaning ability of each coating. The ash was sprinkled on the coated surface, and a water droplet was then placed on the dust^11^. Images were recorded while the water droplet rolled over the dust.

## Results and discussion

### Water contact angle and sliding angle

The wetting properties of a surface are characterized by the contact angle. A high contact angle implies a low wetting of a solid surface. Generally, a surface is hydrophobic when its static water contact angle is greater than 90°, and a surface is classified as superhydrophobic when the contact angle exceeds 150°. In addition, a sliding angle of less than 10° is a self-cleaning surface^6^. The static water contact angle of the coated surface is shown in Table 3.

**Table 3:** Water contact angles and sliding angles of the coated surfaces.

Samples	Contact angle (°)	Sliding angle (°)
Control	113	> 90
CS	140	42.1
CS-F	152	15.8
PTFE	159	8.9
PTFE-F	153	8.7

The results showed that the uncoated geopolymer surface was hydrophilic with a contact angle of less than 90°, which allowed the water to penetrate the matrix. The surface of the PDMS-coated geopolymer (the control sample) was water-resistant and hydrophobic, presenting a contact angle of 113°, similar to that previously reported^33^. However, the above sample was not self-cleaning since it did not have the required surface roughness. Therefore, hydrophobic microparticles, i.e., CS and PTFE, were added to control the geometric structure and increase surface roughness^11,34,35^. Subsequently, the contact angles of water droplets on the coated surfaces increased to 140° and 159° for the coated surfaces containing CS and PTFE, respectively.

With the additional incorporation of FA with CS (CS-F sample), the contact angle was 152°, increasing the superhydrophobicity of the coated surface. This was due to the differences in the particle size and shape of FA and CS, as shown in Table 1 and Fig. 1. The CS particles were deposited over the dispersed FA particles, leading to the formation of small round protuberances. These round bumps, as in the case of lotus leaves^13^, play an important role in conferring superhydrophobic behavior to the coating, preventing wetting and avoiding the penetration of water into concrete.

When FA was additionally incorporated with PTFE (PTFE-F sample), the contact angle was reduced slightly from 159° to 153° compared to only PTFE microparticles. This reduction could be due to the differences in particle shape and size. The grain size of PTFE was much smaller than that of FA. This led to random deposition between the two differently sized types of microparticles. However, the surface was also superhydrophobic. Replacing PTFE with 10% FA in the coating changed the surface from superhydrophobic to hydrophobic when a spray coating method was used^3^. Notably, the dip-coating method used in this study gave good results compared with the spray-coating method^3^. This was due in part to the good dispersion of the microparticles during the sonication process. However, the addition of dispersing agents, such as sodium polyacrylate, sodium hexametaphosphate, and sodium silicate, to the mixture is recommended for industrial applications^36^.

The rolling water test on the coated surface was performed to measure the sliding angle. These results are shown in Table 3. For the control, the PDMS coating provided a hydrophobic surface; however, the sliding angle was over 90°, and the water drop did not roll on this surface. This result was likely because the surface lacked a suitable roughness. When CS microparticles were used, the sliding angle was still high, with a value of 42.1°. This was probably the result of the poor dispersion and agglomeration of particles in the PDMS coating. When FA was incorporated with CS microparticles, the sliding angle decreased to 15.8°, implying an increase in surface roughness. A self-cleaning surface was obtained when PTFE and PTFE/FA microparticles were used. The sliding angles of the PTFE- and PTFE/FA-coated surfaces were similar, namely, 8.9° and 8.7°, respectively. Thus, the coatings incorporating PTFE and PTFE/FA were identified as superhydrophobic and self-cleaning surfaces (≤ 10°), displaying the “lotus effect”^7^.

### Surface analysis

**Figure 3:**
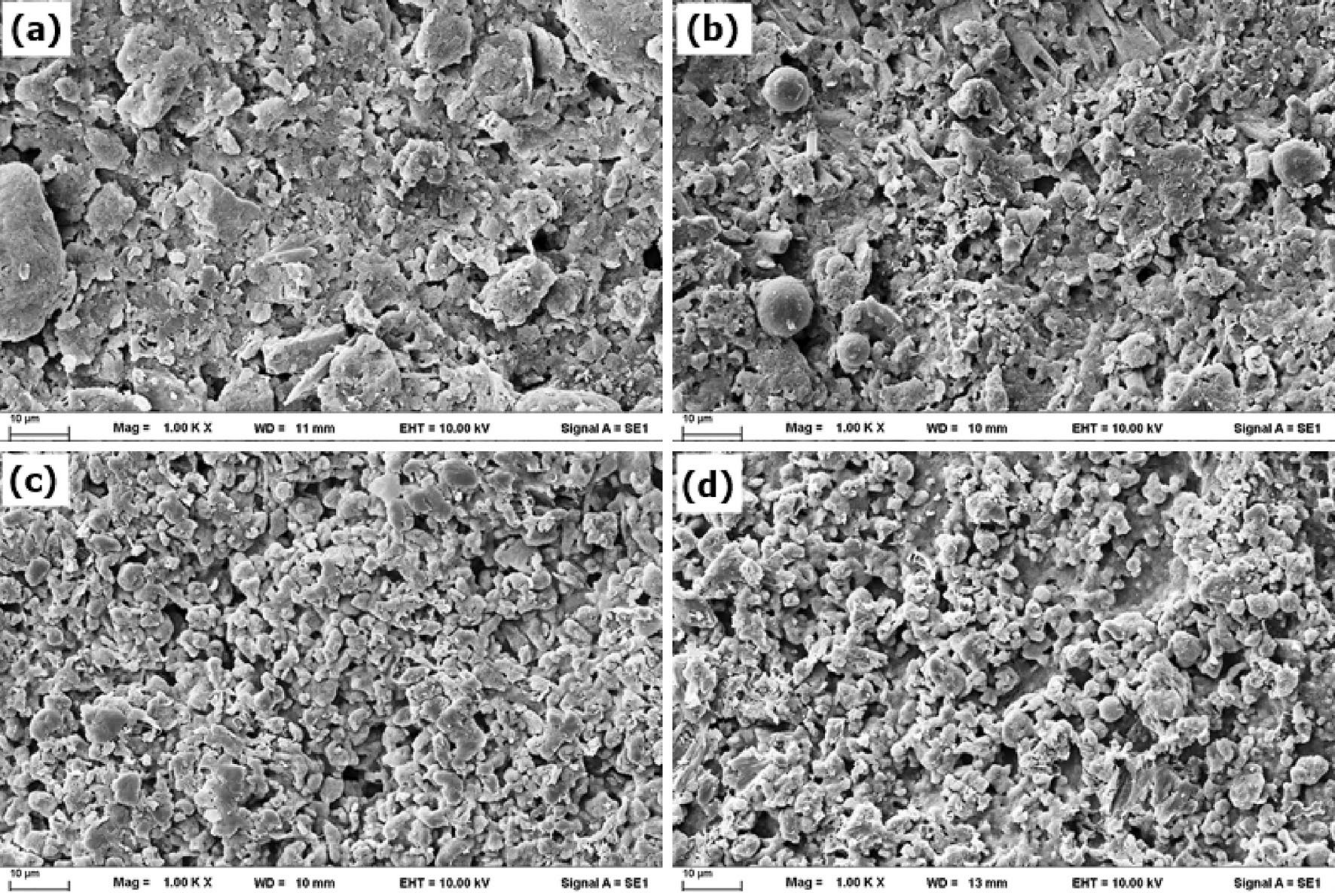
SEM micrographs of the coated surfaces: (**a**) CS, (**b**) CS-F, (**c**) PTFE, and (**d**) PTFE-F.

**Figure 4:**
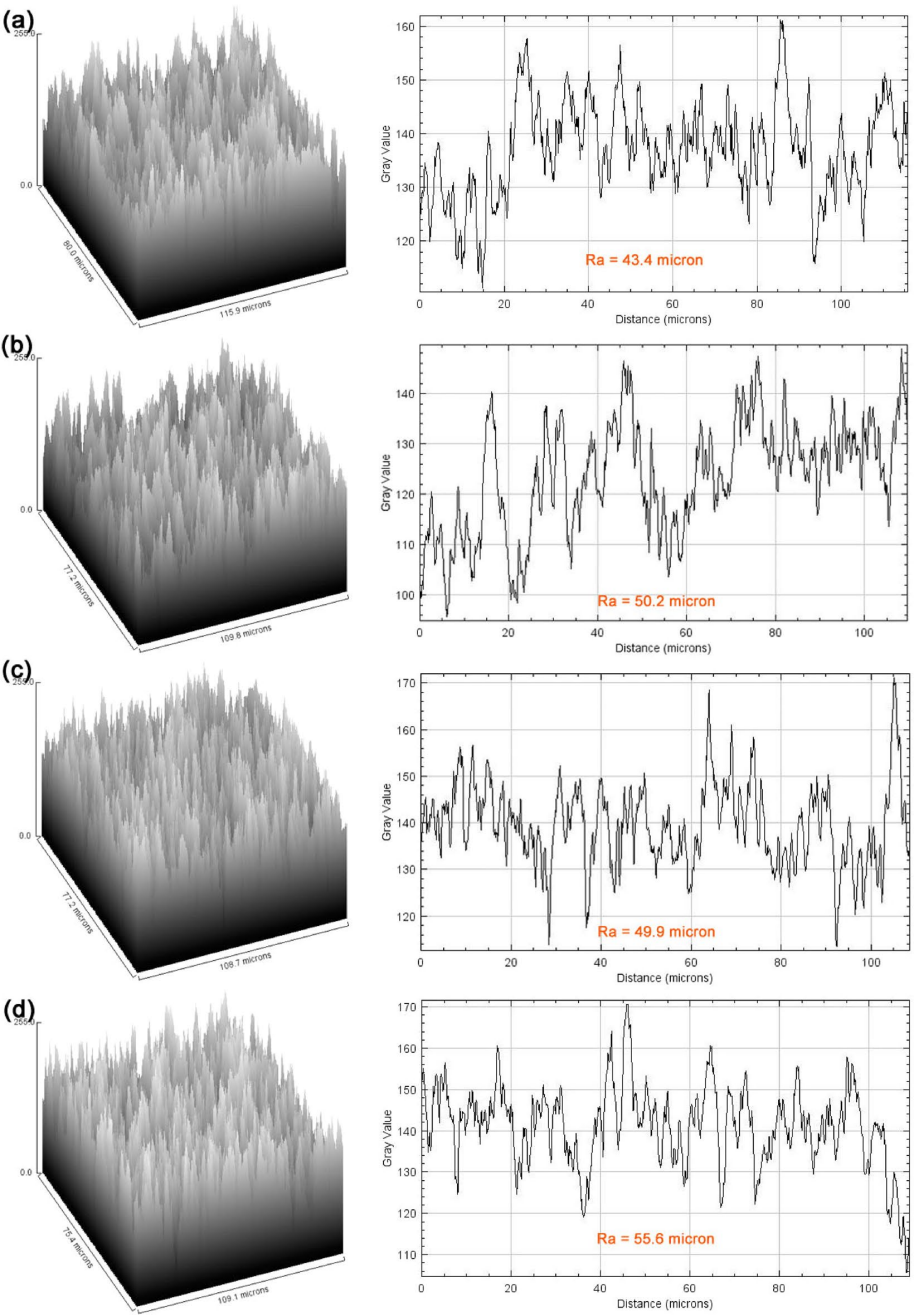
3D images and surface profiles of the coated surfaces: (**a**) CS, (**b**) CS-F, (**c**) PTFE, and (**d**) PTFE-F.

SEM micrographs of the coated surfaces are shown in Fig. 3. Agglomeration was observed in the CS-coated sample (Fig. 3a), which was due to the low dispersion of the CS particles in the PDMS-coated base, whereas the PTFE particles were well dispersed (Fig. 3c). A strong interfacial attraction force also leads to the agglomeration of particles^37^. When FA was blended with CS, a uniform surface with many FA particles was observed (Fig. 3b). For the PTFE-F sample, good blending resulted in a uniform surface containing only a few small FA particles (Fig. 3d). The roughness of the coated surfaces was qualitatively evaluated using the grayscale color of the samples. Roughness plays an important role in the wetting properties of surfaces. An increase in surface roughness results in a larger contact angle^8^. The 3D images and surface profiles from SEM (Fig. 3) are shown in Fig. 4.

Figure 4a shows low and high narrow peaks in the CS sample with bases 5–10 µm wide. In the CS-F sample, the peak base was wider, 10–20 µm, as shown in Fig. 4b, indicating that the fly ash particles were covered by the CS particles, resulting in broader peaks. The same trend was observed in the PTFE-F sample. The uniform roughness of the PTFE-coated surface is shown in Fig. 4c, which agrees with previous research^3^. This effect was due to the small particle size of PTFE and its good dispersion in the PDMS base coating. In addition, the peak heights were approximately the same as those with the addition of FA (Fig. 4d). The Ra values were calculated with the ImageJ program. The Ra values of the CS, CS-F, PTFE and PTFE-F samples were 43.4, 50.2, 49.9 and 55.6 μm, respectively. Compared to the CS or PTFE particles alone, the incorporation of fly ash with CS or PTFE led to a slight increase in the Ra value.

In the case of PTFE with FA, the contact angle and sliding angle of this surface did not change much and still indicated a superhydrophobic and self-cleaning surface. Using two particle sizes is generally required to obtain a superhydrophobic lotus-like surface^38^. However, dosing with 10% by weight of the water-repellent material was recommended because the large particles of FA hindered the formation of lotus-like multiscale structures^3^ and resulting in a sliding angle greater than 10°. A high contact angle surface does not always lead to a low sliding angle, as the roughness of the surface affects the sliding angle. A needle-like structure with a pitch of less than 1 µm was preferred to provide a low sliding angle^6^. Therefore, the incorporation of nanoparticles with microparticles was critical for achieving a low sliding angle on the coated geopolymers.

Coating adhesion tests were conducted according to ASTM D3359-17^30^. The adhesion was rated by inspection of the grid area after tape pull-off, as shown in Table 4. The coating with PDMS (the control) gave a 4B rate with 4% of the area removed, whereas the addition of water-repellent particles into the PDMS resulted in lower adhesion rates of 3B and 2B with a high percent of the area removed, particularly with the 2-microparticle coating. This result could stem from the heterogeneous mixture of the coated solution and separation of the agglomerated microparticles by the blade knife. Therefore, a good dispersion of microparticles and low dilution of PDMS are beneficial for obtaining good adhesion.

**Table 4:** Adhesion results of the coated surfaces.

Samples	% Area removed	Classified under ASTM D3359-17	Images
Control	< 5	4B	
CS	5–15	3B	
CS-F	15–35	2B	
PTFE	5–15	3B	
PTFE-F	15–35	2B	

### Self-cleaning and waterproofing properties of coated surfaces

**Figure 5:**
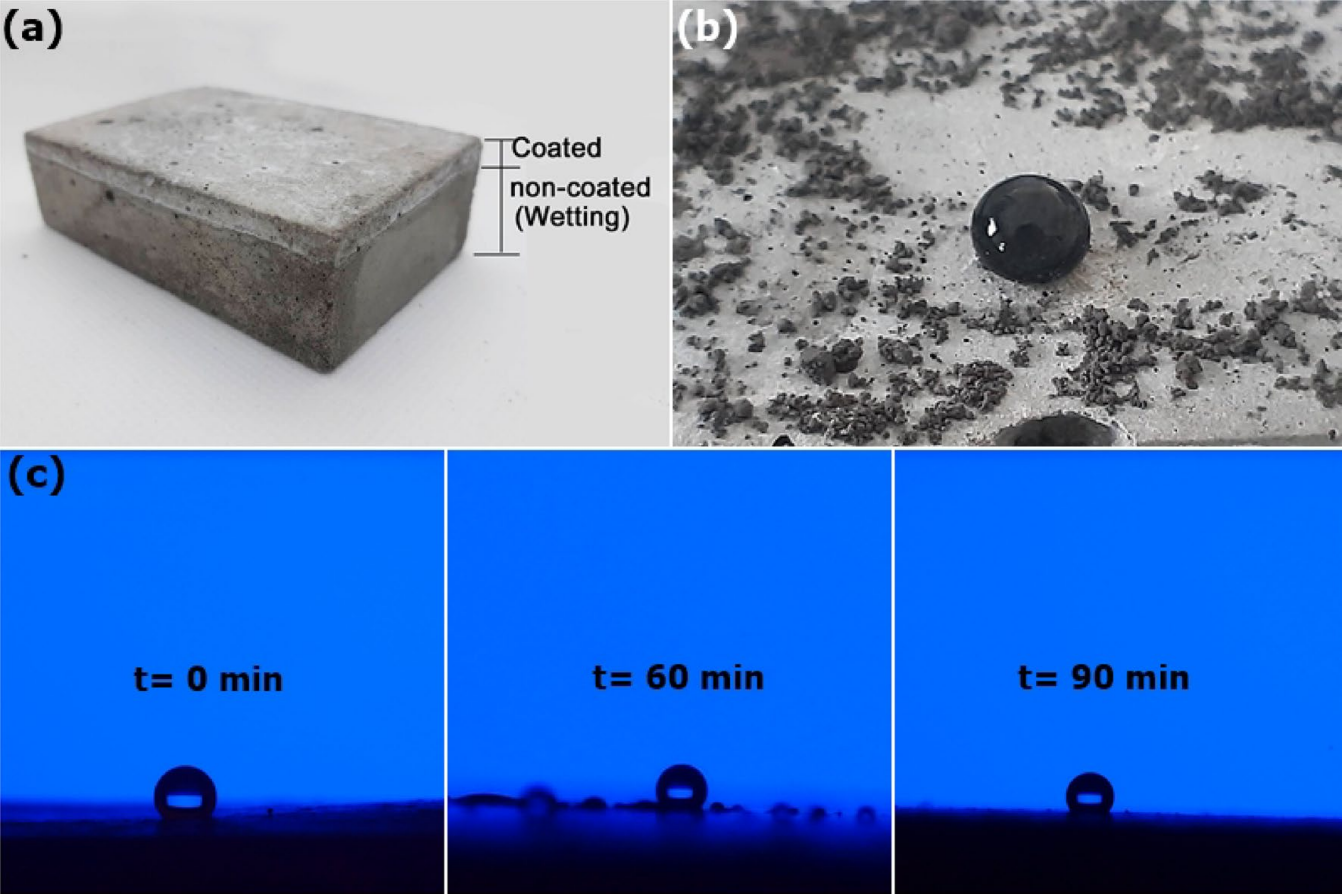
Self-cleaning process and waterproofing ability of the PTFE-F-coated surface: (**a**) coated and noncoated zones, (**b**) dust cleaning, and (**c**) water droplet on a coated surface at t = 0, 60 and 90 min.

The self-cleaning process results from water droplets rolling off the surface and collecting the dust along the way. The self-cleaning process of the coated surfaces is shown in Fig. 5 and Video S1. When the geopolymer mortar surface was partly dip-coated and immersed in water, a wetted zone was observed on the noncoated surface, as shown in Fig. 5a. Generally, the geopolymer or cement concrete surface is hydrophilic, allowing water to easily permeate through and resulting in deterioration of the concrete structure; additionally, if reinforcing bars are present, corrosion may occur. When the surface was coated with a PTFE/FA-based coating, the hydrophobic property of the surface improved. When a water droplet was rolled onto the coated surface covered with dust, the dust was picked up by the water droplet, as evidenced by the lighter path of the droplet (Fig. 5b).

The waterproofing ability of the coated surface was tested by investigating the shape of the water droplet at 0, 60 and 90 min in a 100% relative humidity atmosphere. As shown in Fig. 5c, the round shape of the water droplet was maintained after the 90 min test period. In addition, a video clip of water being sprayed on the coated surface is provided in the supplementary data (Video S2).

The self-cleaning mechanism on a rough surface occurs because water droplets move or roll along the coated surface, and the existing dust is collected into the water droplets. The self-cleaning of dirt particles is possible when the surface roughness minimizes both the adhesion of the droplet to the surface along with minimizing the adhesion between the dirt particles and surface; thus, the dirt particles are easily picked up by the rolling droplets, which is known as the “lotus effect”^7,39^.

Hence, the described coatings incorporating PTFE or PTFE/FA microparticles provide the geopolymer surface with self-cleaning and waterproofing properties. Furthermore, these coatings may reduce dust accumulation and prevent water penetration, prolonging the service life of these building materials. Generally, hydrophobicity is required for building materials under severe conditions, such as concrete structures exposed to marine environments^40^ and concrete in acidic environments^41^. The hydrophobic surface protects the corrosion of steel from chloride ion penetration and the dissolution of calcium from concrete under acidic conditions.

The coating solution could be applied to buildings by painting methods. In this case, for large-scale production, a dispersing agent and uniform mixing are recommended. Dispersion agents, such as sodium polyacrylate, sodium hexametaphosphate, and sodium silicate, have been recommended for industrial applications to improve the dispersion of microparticles^36^. Due to the use of a solvent to dilute PDMS, care and protection are required during the coating process.

## Conclusion

Self-cleaning superhydrophobic surfaces on geopolymer surfaces were fabricated by utilizing dip-coating to achieve a composite coating of PDMS containing dispersed PTFE or CS microparticles mixed with FA. The incorporation of FA with CS or PTFE microparticles was essential to obtain superhydrophobic surfaces due to increasing the surface roughness, which led to a higher water contact angle of 140°–159°. However, the sliding angles of the two systems were divergent. The incorporation of CS resulted in the rose petal effect with a high sliding angle exceeding 90°. The incorporation of FA with the PTFE microparticles resulted in a sliding angle of 8.7°, providing for the self-cleaning properties described as the “lotus effect”. The coated surface was also waterproof, as the water droplet maintained its round shape after the 90 min test period. Therefore, the geopolymer coated with the PDMS composite containing PTFE/FA microparticles could reduce the accumulation of dust and prevent the penetration of water, leading to a clean surface that would prolong the service life of these building materials.

## Supplementary Information

Supplementary Information 1.

Supplementary Video 1.

Supplementary Video 2.

## Data Availability

The datasets used and/or analyzed during the current study are available from the corresponding author on reasonable request.
