# Supplementary material for: Self-cleaning superhydrophobic fly ash geopolymer
Source: Sci Rep. 2023 Jan 2;13:44. doi: 10.1038/s41598-022-27061-6 (PMC9807559; doi:10.1038/s41598-022-27061-6)
Supplement: Supplementary file 1 — Supplementary Information 1. [file 41598_2022_27061_MOESM1_ESM.doc]

**Supplementary data**

For the article “Self-cleaning superhydrophobic fly ash geopolymer”

By Prinya Chindaprasirt, Peerapong Jitsangiam, Pumipat K. Pachana and Ubolluk Rattanasak

**Video S1** Self-cleaning process of coated surface

**Video S2** Video clip of water spraying to coated surface
